# Integrating Transcriptomic and Proteome Analysis to Understand Muscle Qualities and Related Regulatory Factors of Holstein Beef Under Transportation Stress

**DOI:** 10.1002/fsn3.72415

**Published:** 2026-10-12

**Authors:** Zhaomin Lei, Siyu Cheng, Ruixin Yang, Jinping Shi, Zemin Li, Wangjing Liu

**Affiliations:** ^1^ College of Animal Science and Technology Gansu Agricultural University Lanzhou China; ^2^ China Resources Ng Fung, Ltd Shenzhen China; ^3^ College of Animal Sciences and Technology Shandong Agricultural University Taian China

**Keywords:** data‐independent acquisition‐proteomics, flavor, meat quality, RNA‐sequencing, transport stress

## Abstract

This study aimed to explore the impact of transportation stress on beef quality and related regulatory mechanisms. Twelve Holstein steers of similar weight were divided into two groups: transportation for 15 h (STR) and transportation for 0 h (CON). The differences in meat quality, myofibril structure, and flavor components between transport‐stressed and normal beef were compared. Through a comprehensive analysis of the transcriptome and proteome of the *longissimus lumborum*, the genes, proteins related to transport stress in meat were identified. The study found that the shear force, water loss rate, and drip loss of beef in the STR group were significantly higher than those in the CON group (*p* ≤ 0.05), and the marble grade of beef in the STR group was significantly lower than that in the CON group (*p* = 0.035); Muscle transmission electron microscopy, it was found that transportation significantly shortened the length of the sarcomere. Furthermore, long‐distance transport has a detrimental effect on muscle flavor. We found that six DEGs/DEPs showed the same trend in mRNA and protein expression. It is plausible that transport stress influences beef quality and flavor, at least in part, through ferroptosis pathway‐mediated lipid peroxidation. Meanwhile, the observed changes in stress‐responsive genes/proteins (HMOX1 and BDH1) were linked to concomitant alterations in meat quality‐associated genes/proteins (COL1A1, FMOD, and MYL3), indicating that these molecular events may collectively contribute to quality deterioration. Our results provide important insights into the impact of transportation stress on beef quality.

## Graphical abstract

In Holstein steers subjected to 15 h of long‐distance transportation, beef exhibited marked differences in meat quality, myofibrillar structure, and flavor components compared with cattle slaughtered without prior transport. These differences included significantly increased shear force, water loss rate, and drip loss, shortened sarcomere length, and adverse effects on flavor. The ferroptosis pathway and stress‐related genes/proteins (HMOX1 and BDH1) were identified as key pathways and molecular mediators through which transport stress affects meat quality.

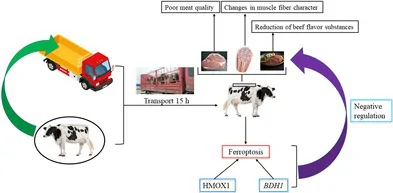

## 1 Introduction

In the 1990s, developed countries such as Europe and the United States had already started using dairy bulls as one of their main sources of beef (Wilson et al. 1999). For example, 45% of beef consumed in EU countries comes from dairy bulls, while 30% of beef produced in the United States comes from dairy bulls; 90% of Dutch beef comes from dairy calves. Hence, dairy bulls constitute a notable supplemental contribution to overall beef output. In order to reduce feeding costs and financial investment in dairy farms, countries such as South Korea, the United States, and the Netherlands all use 16–19 week old male dairy calves as meat and rarely fatten them (Cho et al. 2014; Ngapo and Gariépy 2006), which has gradually become a trend for male dairy beef. However, despite decades of dedicated breeding, dairy bulls still exhibit inferior meat production performance compared with conventional beef cattle (Corazzin et al. 2011; Rezagholivand et al. 2020), although their meat quality and flavor have been continuously improved. Wang et al. (2005) used gene chip technology to compare and analyze the gene expression profiles of the *longissimus lumborum* (LL) between Japanese black cattle and Holstein cattle at different growth stages and found that Holstein cattle had the molecular basis for producing high intramuscular fat, but the developmental stage lagged behind that of Japanese black cattle. Therefore, proper fattening of dairy bulls is an important way to improve meat quality and increase beef production.

In modern animal husbandry, long‐distance transportation of animals is often unavoidable. This involves transporting animals from a farm to a saleyard, market, or abattoir, or from a farm specializing in breeding stock to a farm specializing in fattening. For example, in China, approximately 50 million cattle are transported to different places for slaughter every year, and the stress reactions generated during transportation have a direct impact on beef quality (Bulitta et al. 2015). Such transportation processes can span thousands of kilometers and take several days. During transportation, feed and water are typically withheld to reduce digesta load in the digestive tract, minimize contamination of other animals, transport vehicles and roadways, and enable accurate carcass weight assessment (Hogan et al. 2007). However, these practices can induce stress in animals and have significant adverse impacts. There are reports that transportation duration to slaughterhouses should not exceed 16 h (Mota‐Rojas et al. 2006). Transportation stress refers to the adaptive and defensive response of the animal body caused by the combined effects of various stressors such as fasting, environmental changes, physical, and psychological stress during transportation (Knowles et al. 2014). After long‐distance transportation, ruminants may experience a decrease in rumen motility, abnormal fermentation, and unstable rumen pH, which directly affects abnormalities in the microbial flora in the rumen (Adams‐Progar et al. 2015), leading to weight loss, elevated body temperature, decreased food intake, and reduced or ceased rumination (González et al. 2012). When animals are in a stressed state prior to slaughter, it leads to elevated metabolic activity in the muscles both during and after the slaughter process. Muscle glycogen becomes substantially depleted. This results in pH decline while the carcass is still warm, leading to PSE (pale, soft, exudative) meat formation. The combination of low pH and elevated temperature induces muscle protein denaturation, resulting in pale‐colored meat with poor water‐holding capacity and excessive drip loss (Kumar et al. 2022). Furthermore, prolonged transportation exacerbates muscle contraction intensity, with myofibrillar shrinkage propagating throughout the entire muscle cell and inducing intermyofibrillar space enlargement (Xing et al. 2022). For pigs slaughtered immediately after transport stress, the water‐holding capacity of the muscles decreases, and the incidence of abnormal meat increases. Slaughtering after a 2–3 h rest allows pigs to recover better from stress, significantly reducing the aforementioned adverse effects (Ma, Zhang, and Xing 2024). Currently, research on the impact of long‐distance transportation on animals is relatively concentrated in routine studies such as blood physiology and biochemistry (Mitchell and Kettlewell 1999), food intake (Adams‐Progar et al. 2015), and mortality (Lewis et al. 2010). Research on the impact of meat quality has also focused on tenderness, pH (Lacerda et al. 2022), and meat color (Van de Water et al. 2003). There are few integrated transcriptome and proteome studies on the effects of transportation on quality regulatory factors in dairy male beef. Post‐translational modifications, degradation of myofibrillar proteins, and oxidative modifications of myoglobin constitute key biochemical determinants of meat quality. Muscle proteomics provides a valuable approach for characterizing meat quality defects. Comprehensive proteomic profiling of muscle tissues enables an in‐depth understanding of molecular mechanisms affected by pre‐slaughter stress across different stages of animal production (Marco‐Ramell et al. 2016).

In this study, we explored the following two basic issues using transcriptomics and proteomics together with research on meat quality, beef flavor compounds, and muscle tissue morphology: does long‐distance transportation affect meat quality‐related regulatory factors, and how are these regulatory factors affected? This study provides molecular information on the impact of transportation stress on meat quality‐related factors, the identification of which will enhance quality in dairy steer production.

## 2 Materials and Methods

### 2.1 Animals, Feeding Management, and Experimental Design

The experiments were conducted at Huarui Ranch, Minle County, Zhangye, Gansu Province. Forty healthy Holstein dairy bulls with insignificant weight differences were selected, and they were castrated at the age of 2 months. They were raised to 10 months of age under the same nutritional level and management conditions. Eighteen steers with similar body weights (351.62 ± 19.88 kg) were randomly selected from the cohort and subsequently housed individually for a period of 9 months. During the experiment, animals were fed a total mixed ration (TMR) consisting of corn, silage, and a mixture of grains. During the experiment, all animals were allowed to freely ingest feed and free water, ensuring the same nutritional level and management conditions. On Day 270 of the trial, 12 healthy steers with similar body weights (636.63 ± 47.31 kg) were selected from the 18 experimental cattle and, using a randomized complete block design with body weight as the blocking factor, were randomly allocated to two groups (*n* = 6 per group): a 0‐h transport control group (CON) and a 15‐h transport stress group (STR). The CON group continued to raise at Huarui Ranch, Minle County, Zhangye, Gansu Province. The STR group was transported using one truck (truck compartment: 4.5 × 1.8 m) to Gansu Xukang Food Co. Ltd. (Pingliang, Gansu, China). The top of the carriage was covered with tarpaulin. A total distance of 892 km was covered at an average speed of 75 km/h, such that the trip lasted 15 h and 24 min. During transportation, the CON group was also denied access to food and water. At 08:00 the following morning, cattle from the STR group and CON group were slaughtered concurrently within a one‐hour timeframe at two separate abattoirs: animals of the STR group were processed at the abattoir in Pingliang, while the CON cattle were slaughtered at the abattoir in Zhangye. Identical standardized slaughtering procedures were carried out at the two sites. All bovines were stunned using an electrical captive bolt device (220 V, 1.0–1.5 A), followed by full exsanguination via severance of the jugular vein.

### 2.2 Sample Collection

A total of 12 cattle, comprising 6 animals in the CON group and 6 in the STR group, were used for muscle tissue sampling. LL muscle samples were collected from the left carcass side at the region between the 10th and 13th ribs and were subsequently subjected to integrative transcriptomic and proteomic analyses. The LL samples collected from each animal were immediately frozen in liquid nitrogen and stored at −80°C until further use. Subsequently, the carcass was suspended in the chiller at the slaughterhouse for 24 h; the conditions in the chillers were the same (temperature, 0°C–4°C; humidity, 85%; wind speed, 2–4 m/s). Two portions of muscle tissue were taken from the LL on the left side of each steer. One portion was fixed in glutaraldehyde solution and stored at 4°C for transmission electron microscope (TEM) scanning. The other portion was stored at −80°C for flavor substance determination.

### 2.3 Meat Quality Assessment

All meat quality traits were measured in triplicate unless otherwise specified. The pH of the muscle tissue was determined at 24 h postmortem using a portable pH meter (PHS‐10, Fangzhou Technology, Chengdu, China), with the electrode inserted directly into the meat for 30–60 s until a stable reading was obtained, after calibration at 20°C with pH 4.01 and 6.86 buffer solutions. Color coordinates (lightness L, redness a, yellowness b*) were measured with a colorimeter (Matthaus GmbH & Co. KG, Eckelsheim, Germany) equipped with a D65 illuminant, a 10° standard observer, and a 26‐mm aperture, taking readings at three random positions per sample. Water loss rate was assessed by the filter‐paper press method: muscle cores taken with a circular sampler and cut into 1‐cm‐thick slices were weighed to obtain the initial mass (W_1_), wrapped in gauze, sandwiched between 18 layers of high‐absorbency filter paper on each side, pressed under a 35‐kg load for 5 min, and immediately reweighed (W_2_); the loss percentage was calculated as (W_1_ – W_2_)/W_1_ × 100. Drip loss was determined on 2 × 2 × 4 cm muscle strips, which were weighed (W_1_), suspended vertically inside plastic bottles using hooks to avoid wall contact, and stored at 4°C for 24 h; after storage, the strips were blotted dry with filter paper and reweighed (W_2_), with the loss expressed by the same formula. Cooking loss was evaluated on 2.5‐cm‐thick samples, weighed (W_1_), sealed in heat‐resistant vacuum pouches, and immersed in a thermostatically controlled water bath at 80°C until the geometric centre reached 70°C; the cooked samples were then cooled to room temperature, surface‐dried with filter paper, and reweighed (W_2_), and the loss was calculated using the identical percentage equation. Shear force values were measured using a Warner–Bratzler apparatus on 1 × 1 cm strips cut parallel to the muscle fiber direction, with three replicate readings obtained per strip. Marbling scores were determined according to the United States Department of Agriculture (USDA) quality grade standards (U.S. Department of Agriculture and Agricultural Marketing Service 1997), with the evaluation performed by trained panelists on the cut surface of the LL muscle at the 12th–13th rib interface at 24 h postmortem; marbling was assigned one of six grades—a (moderately abundant), b (slightly abundant), c (moderate), d (modest), e (small), and f (slight)—which were subsequently converted to numerical scores (a = 6, b = 5, c = 4, d = 3, e = 2, f = 1) for statistical analysis.

### 2.4 Observation of Muscle Tissue Using TEM

All experimental animals were slaughtered and the LL samples were collected and pre‐fixed with 3% glutaraldehyde and re‐fixed with 1% osmium tetroxide. Step by step dehydration of acetone (30%, 50%, 70%, 80%, 90%, 95%, and 100%) was performed. After passing through the dehydrating agent and epoxy resin penetration liquid, the permeated sample block was placed in a suitable mold, filled with embedding liquid, and then polymerized at elevated temperatures to form a solid matrix. Ultrathin slices with a thickness of 50 nm were prepared using an ultrathin sectioning machine (Hengsong, Shengyang, Chian). The samples were dyed with uranium acetate for 10–15 min and lead citrate for 1–2 min. The sectioned samples were observed using a JEM‐1400PLUS TEM (JEOL, Tokyo, Japan). The JEM‐1400FLASH (JEOL, Tokyo, Japan) TEM (Japan Electronics) was used for image acquisition.

### 2.5 Volatile Flavor Compound Analysis

The volatile flavor compounds in the LL meat samples were analyzed using a GC–IMS (FlavourSpec, G.A.S., Doetmund, Germany) equipped with an MXT‐5 capillary column (Restek, PA, USA) and an autosampler (CTC Analytics AG, Zwingen, Switzerland) with a headspace sampling unit and a gas‐tight syringe (Gerstel GmbH, Mühlheim, Germany). Briefly, the ground meat samples were thawed overnight in a refrigerator (12 h at 4°C) and 3 g of sample was accurately weighted and placed in a 20 mL headspace (HS) (HM‐2075G, HAMAG, Ningbo, Zhejiang, China) vial with a magnetic screw seal cover (HM‐2075G, HAMAG, Ningbo) followed by incubation at 60°C for 15 min. Then, 500 μL of HS gas was injected automatically with an injector temperature set at 70°C using a splitless injection. The column was maintained at 60°C and the drift tube temperature at 45°C. The drift gas was nitrogen at a flow rate of 150 mL/min. The carrier gas flow rate was set to 2 mL/min for first 2 min, repeated once, and then increased to 100 mL/min for 16 min, and then the flow rate was stopped. Each group consisted of six samples, and each sample was measured in triplicate. The mean of the three technical replicates was used as the final value for subsequent analyses. The retention index (RI) was calculated using n‐ketones C4–C9 (Sinopharm Chemical Reagent Beijing Co. Ltd., Beijing, China) as external reference standards. The volatile flavor compounds were identified by comparing RI and Dt (drift time) of the GC–IMS library standards. Dt was defined as the time required for ions to reach the collector through the drift tube. The volatile flavor compounds were calculated based on the peak height of the identified signal peaks (Liu et al. 2020).

### 2.6 cDNA Library Preparation and RNA‐Seq

TransZol (Transgen Biotechnology, Beijing, China) was used to extract total RNA from muscle samples. RNA concentration was measured using a Qubit RNA Assay Kit in a Qubit 2.0 Fluorometer (Life Technologies, CA, USA). RNA integrity was assessed using the RNA Nano 6000 Assay Kit of the Bioanalyzer 2100 system (Agilent Technologies, CA, USA) (Shi et al. 2022). One microgram of RNA was extracted from each sample for library construction. Briefly, mRNA was purified from total RNA using poly‐T oligo‐attached magnetic beads. Fragmentation was carried out using divalent cations under elevated temperature in NEBNext First Strand Synthesis Reaction Buffer (5×). First strand cDNA was synthesized using random hexamer primer and M‐MuLV Reverse Transcriptase (RNase H). Second strand cDNA synthesis was subsequently performed using DNA Polymerase I and RNase H. The library fragments were purified with the AMPure XP system (Beckman Coulter, Beverly, USA). Then, 3 μL USER Enzyme (NEB, MA, USA) was used with size‐selected, adaptor‐ligated cDNA at 37°C for 15 min followed by 5 min at 95°C before PCR. Then PCR was performed using Phusion High‐Fidelity DNA polymerase, universal PCR primers, and Index (X) primer. PCR products were purified using the AMPure XP system and library quality was assessed on the Agilent Bioanalyzer 2100 system. The constructed cDNA libraries were sequenced on an Illumina Novaseq platform (Beijing, China) (Roberts et al. 2011; Zhao et al. 2022).

### 2.7 Quality Control, Annotation, and Differential Expression Analysis

The original reading material was filtered and purified by removing the containing adapter, POLY‐N (the percentage of uncertain N greater than 10%), and‐low quality reads (reads containing > 50% bases with a Q ≤ 20). The clean reads were compared with the beef cattle genome. Based on the raw integer count data, differential expression analysis of genes was performed using the DESeq2 package (version 1.16.1). The Benjamini–Hochberg method was applied for multiple testing correction of the original *p*‐values generated by DESeq2 to obtain false discovery rate (FDR)‐adjusted *p*‐values. Genes with an FDR value of less than 0.05 and an absolute log_2_ (fold change) value of no less than 1 were defined as differentially expressed genes. Transcripts per million (TPM) values were computed for expression visualization according to established normalization procedures (Wagner et al. 2012).

### 2.8 Protein Extraction and Mass Spectrometry Detection

After fully grinding the muscle tissue, lysate (1.5% SDS/100 mM Tris‐Cl) was added to the sample, fully mixed, and centrifuged to obtain the supernatant. The protein in the supernatant was precipitated using the acetone precipitation method. The protein precipitate was dissolved using a complex solution (8 M Urea/100 mM Tris‐Cl), and incubated at 37°C for 1 h with dithiothreitol. Subsequently, iodoacetamide was added to induce an alkylation reaction to block the sulfhydryl group. The protein concentration was measured using the Bradford method. An amount of 100 mM Tris–HCl solution was added to the sample. The urea concentration was diluted to below 2 M. The trypsin was added according to the mass ratio of enzyme to protein (1:50), incubated at 37°C, and shaken overnight for enzymatic digestion. The next day, TFA was added to terminate the enzyme digestion, and the supernatant was taken for desalination.

Mass spectrometric data for each desalted sample were collected using an Orbitrap Explorer 480 mass spectrometer (Thermo Fisher Scientific, Cambridge, MA, USA) in series with an EASY‐nLC 1200 liquid phase liquid mass spectrometry system. The peptide sample was dissolved in a loading buffer, inhaled by an automatic sampler, and then combined with an analytical column (75 μm*25 cm, C18, 1.9 μm, 100 Å) for separation. An analytical gradient was established using two mobile phases (mobile phase A: 0.1% formic acid and mobile phase B: 0.1% formic acid, 80% ACN). The liquid phase flow rate was set to 0.3 μL/min. For Data‐Dependent Acquisition (DDA) analysis, each scan cycle comprised one full‐scan mass spectrum (resolution: 60,000; AGC target: 300%; maximum injection time: 20 ms; scan range: 350–1800 m/z) followed by 20 data‐dependent MS/MS scans (resolution: 15,000; AGC target: 100%; maximum injection time: auto; cycle time: 2 s). The HCD collision energy was set to 30%, and an isolation window of 1.2 Da was used for precursor selection. A dynamic exclusion duration of 35 s was applied for previously fragmented precursor ions. For Data‐Independent Acquisition (DIA) analysis, each scan cycle consisted of one full‐scan mass spectrum (resolution: 60,000; AGC target: 3e6; maximum injection time: 30 ms; scan range: 350–1250 m/z) followed by 40 variable‐window MS/MS scans (resolution: 30,000; AGC target: 1000%; maximum injection time: 50 ms). The FAIMS interface was operated with a compensation voltage of −45 V, and the HCD collision energy was set to 30%.

### 2.9 Offline Data Analysis

DIA raw data were processed using DIA‐NN software (v1.7.16). A hybrid spectral library was constructed: the deep learning module of DIA‐NN predicted spectral profiles based on the UniProt bovine protein database (Uniprot.Proteome.Bovine.20200629.fasta), and spectral information from the match‐between‐runs (MBR) function was integrated to form the final hybrid library for precursor ion extraction (The UniProt Consortium 2008). Identified peptides and proteins were filtered with a strict threshold of 1% FDR at both precursor (peptide) level and protein level, and globally normalized protein quantitative intensities were obtained after filtering. BLAST comparisons (BLAST, evaluation ≤ 1e‐5) were used to annotate GO and KEGG databases. Fold changes of ≥ 1.5 and *p* < 0.05 were considered to indicate differential proteins (DEP). Orthogonal partial least squares discriminant analysis (OPLS‐DA) was performed on the global‐normalized protein abundance matrix to discriminate between the two experimental groups. Prior to OPLS‐DA, the data were log‐transformed and subsequently subjected to unit variance (UV) scaling, in which each variable was mean‐centered and divided by its standard deviation. The OPLS‐DA model was constructed using the two‐group classification (CON vs. STR) as the Y‐response matrix. Model performance was evaluated by the cumulative goodness‐of‐fit parameter R^2^Y(cum) and the cumulative goodness‐of‐prediction parameter Q^2^(cum), which was obtained from seven‐fold cross‐validation. To assess overfitting and validate model robustness, a permutation test with 200 random permutations was performed. The model was considered valid when the Q^2^‐intercept from the permutation test was negative and all permuted Q^2^ values were lower than the original Q^2^ value.

### 2.10 Quantitative Real‐Time PCR Assays for Target Genes

TransZol (Transgen Biotech, Beijing, China) was used to extract total RNA from the LL. Reverse transcription into cDNA was followed by quantitative real‐time polymerase chain reaction (qPCR). The specific primers for the selected genes were designed using Oligo 7.0 and Primer 5 software (Table 1). The qPCR was performed on an ABI7300 real‐time system (ABI system, Foster City, California, USA) using a 20 μL reaction volume and 1 μL cDNA. The SYBR premix Ex TaqII and specific primers were used for each reaction. The expression of the housekeeping gene, *ACTB* and *GAPDH* was used as an in‐group control. A denaturation step was run for one cycle at 95°C for 30 s. The annealing step was run for 40 cycles at 95°C for 5 s and 60°C for 31 s. All PCR reactions were performed in triplicate. The results were calculated using the 2^−△△CT^ method (Livak and Schmittgen 2001; Shi et al. 2021).

**TABLE 1:** Parameters of primer pairs used for qRT‐PCR of Holstein *longissimus lumborum* samples.

Gene name	Annealing temperature (°C)	Primer sequences (5′–3′)	Product length (bp)
*COL1A1*	F: ATGTGCCACTCTGACTGGAA	55.6	170
	R: TCCTTGGGGTTCTTGCTGAT		
*HSPB1*	F: TCACTCGCAAATACACGCTG	58	142
	R: AAGGTGACGGGAATGGTGAT		
*BDH1*	F: CAAAGCTGTCCTGATCACGG	58.6	121
	R: CCCATCACTCCCTTTGTCCT		
*SLC16A3*	F: ACCGTGGCTTCGTCATCTAT	57	158
	R: AGCAAAGATGTCGACGAAGC		
*FMOD*	F: GACCTGAGCTACAACCACCT	53.8	164
	R: TTGTTGGTGAGGCTGTTGTG		
*HMOX1*	F: ATGCCCCAGGATTTGTCAGA	57.6	142
	R: GGTACAAAGACGCCATCACC		
*MYL3*	F: GACTTTGACACGTTCCTGCC	55.5	170
	R: TCTGTCAGCTTCTCACCCAG		
*PFN2*	F: CTACTGCGACGCCAAATACG	58	141
	R: CACTTCTTTGCGCCAAGAGT		
*GAPDH*	F: AAGGCCATCACCATCTTCCA	56–60	158
	R: GGTGCAGAGATGATGACCCT		
*ACTB*	F: CCCTGGAGAAGAGCTACGAG	55–60	129
	R: TAGTTTCGTGAATGCCGCAG		

Abbreviations: ACTB, actin, beta; *BDH*1, 3‐hydroxybutyrate dehydrogenase 1; *COL1A1*, collagen Type I alpha 1 chain; *FMOD*, fibromodulin; GAPDH, glyceraldehyde‐3‐phosphate dehydrogenase; *HSPB1*, heat shock protein family B (small) member 1; *MYL3*, myosin light chain 3; *PFN2*, profilin 2; *SLC16A3*, solute carrier family 16, member 3.

### 2.11 Statistical Analysis

All data were subjected to independent sample *t*‐tests and Pearson correlation coefficient calculations using SPSS version 22.0 (IBM Corp., Armonk, New York, USA). The data are expressed as mean ± SE. *p* ≤ 0.05 was considered statistically significant. ImageJ (National Institutes of Health, Bethesda, MD, USA) was used to count sarcomere length and myofibril diameter. The StringDB server was used for protein–protein interactions (PPI) network analysis, and visualization was performed using Cytoscape software. OriginPro 9.1 (OriginLab, US, USA) and OmicShare tools (https://www.omicshare.com/) were used to draw statistical charts.

## 3 Results and Discussion

### 3.1 Analysis of Meat Quality and Myofiber Histomorphology

This study measured the meat quality indicators postmortem aging for 24 h (Table 2). Muscle characteristics related to meat quality are regulated by age, nutrition, genetics, and environmental factors, which in turn affect the structure, composition, and intramuscular fat of muscle fibers (Bongiorni et al. 2016). Animal transportation is inevitable during livestock breeding, so what effects does transportation have on meat quality, and what are the underlying mechanisms? This study revealed that muscle L*, a*, and b* values after long‐distance transport were significantly decreased compared to the CON group (*p* ≤ 0.01). According to research by Owens and Sams (2000) the L* value of turkey breast muscle decreased after 3 h of transport compared to non‐transported turkeys. Bianchi et al. (2006) found that the a* value of broiler breast meat color was higher following short‐distance transport compared to long‐distance transport, indicating a lighter meat color in broilers subjected to prolonged transit. These results are consistent with the findings of the present study. Cattle under chronic stress conditions (such as maintaining high levels of nervous tension and fasting and water deprivation during transportation) have an increase in the secretion of cortisol (Dixit et al. 2001), resulting in a large amount of decomposition of the liver glycogen stored in the body. Muscle glycogen also actively participates in the energy metabolism of the body when liver glycogen is largely decomposed. However, muscle glycogen does not directly decompose into glucose, but rather decomposes into lactic acid first, which enters the liver through blood circulation to form liver glycogen. During this process, the concentration of lactic acid in muscle and blood will increase, resulting in a downward trend in the pH value of muscle. Severe stress can have a negative impact on the quality of cold and fresh meat even if beef cattle undergo acid removal treatment after slaughter (Gruber et al. 2009). In addition, research has shown that stress‐related elevation of cortisol levels causes a significant decrease in intramuscular fat percentage and rib fat, which in turn diminishes marbling scores (Tan and Jiang 2024). These factors may be associated with the lower marbling scores observed in the STR group in the present study. The rate and extent of postmortem pH decline, particularly during the early period following slaughter, critically influence key meat quality traits, including water‐holding capacity, tenderness, and color development. Deviations from the normal glycolytic flux and the attendant pH decrease are consistently associated with impaired meat quality (Ma et al. 2025). For instance, rapid glycolysis leading to an abrupt pH drop gives rise to PSE meat, a condition frequently observed in pork and poultry. Conversely, insufficient glycolytic activity and retarded pH reduction are linked to other quality defects, such as the high prevalence of wooden breast in chicken and dark, firm, and dry (DFD) meat in beef (Liao et al. 2023; Ma, Zhang, Zhang, et al. 2024). Collectively, these examples underscore that both excessive and suppressed postmortem glycolysis adversely affect the ultimate meat properties. Studies have demonstrated that the muscle pH of most animals declines from an initial value of approximately 7.0 to an ultimate pH of 5.5–5.7 within 24 h postmortem. In the present study, the ultimate pH of the CON group was 5.89, which was very close to this physiological range, while the STR group also fell within the aforementioned interval. These results may tentatively suggest that transport‐induced stress might not be severe enough to induce PSE or DFD meat defects. Our results provide evidence for the aforementioned conclusion. Furthermore, pH value influences meat color, tenderness, texture, and water‐holding capacity. A lower pH promotes the denaturation of muscle proteins, which in turn reduces water‐holding capacity and leads to increased purge loss (drip loss) from the muscle surface (Gou et al. 2021). This is consistent with the results of this study that transport stress increases drip loss and water loss rate. Due to the many pre‐slaughter factors that affect beef quality, such as pre‐slaughter fasting and water restriction, transportation, and lairage times, different studies have reached inconsistent conclusions. For example, Fernandez et al. (1996) believe that long‐distance transportation of beef cattle for 11 h substantially reduced the glycogen content, glycolytic potential, and postmortem pH of the LL, but had no marked impact on other qualities, such as water loss rate and cooking loss. The current study found that the shear force in the CON group was lower than that in the STR group (*p* ≤ 0.01), and the drip loss and water loss rate were lower than those in the STR group (*p* ≤ 0.05). The main structural component of the meat is the myofibrils, which account for 70% of the volume of lean meat. Myofibrils contain 20% protein, and the rest is water. Most of the water in meat is present in myofibrils between the coarse and fine fibers (Offer and Trinick 1983). Therefore, changes in water retention of meat are likely caused by changes in the volume of myofibrils, and these changes are caused by changes in filament spacing. The contraction of myofibrils causes water to gradually move from within the myofibrils to outside the myofibrils, resulting in increased drip loss (Hughes et al. 2014). In addition, the contraction of myofibrils shortens the sarcomere and increases the shear force, resulting in poorer tenderness (Herring et al. 1965). In this study, the cooking loss of the STR group was lower than that of the CON group (*p* = 0.006). The results of the TEM scanning of muscle fibers showed that there was a marked difference between the CON group and the STR group in postmortem aging for 24 h (Figure 1A), and the degree of myofibril autolysis in the STR group was substantially higher than in the CON group. In order to compare the differences more intuitively in postmortem aging for 24 h, a statistical analysis of sarcomere length and myofibril diameter was conducted. The sarcomere length of the CON group was greater than that of the STR group (*p* = 0.000), and there was no difference in myofibril diameter between the two groups (*p* = 0.334; Figure 1B). This finding is consistent with the results of drip loss and water loss rate, indicating that transport stress alters the muscle fiber structure and impairs the water‐holding capacity of the muscle. Collectively, transport stress adversely affects both meat quality and myofiber structure.

**TABLE 2:** Difference in meat quality of Holstein steers transported for 15 h and transported for 0 h.

Items	CON	STR	Significance
Marbling score	4.00 ± 0.37	2.50 ± 0.31	*
pH	5.89 ± 0.11	5.52 ± 0.03	*
L*	26.68 ± 0.01	26.04 ± 0.71	**
a*	20.07 ± 0.25	18.84 ± 0.18	**
b*	7.24 ± 0.19	6.15 ± 0.36	**
Shear force (Kgf)	10.44 ± 0.16	12.59 ± 0.30	**
Cooking loss (%)	21.84 ± 0.29	20.32 ± 0.32	**
Water loss rate (%)	17.34 ± 0.29	19.27 ± 0.67	*
Drip loss (%)	0.42 ± 0.01	0.72 ± 0.02	*

Abbreviations: a*, redness; b*, yellowness; CON, transportation for 0 h; L*, lightness; STR, transportation for 15 h.

* *p* < 0.05.

** *p* < 0.01.

**FIGURE 1:**
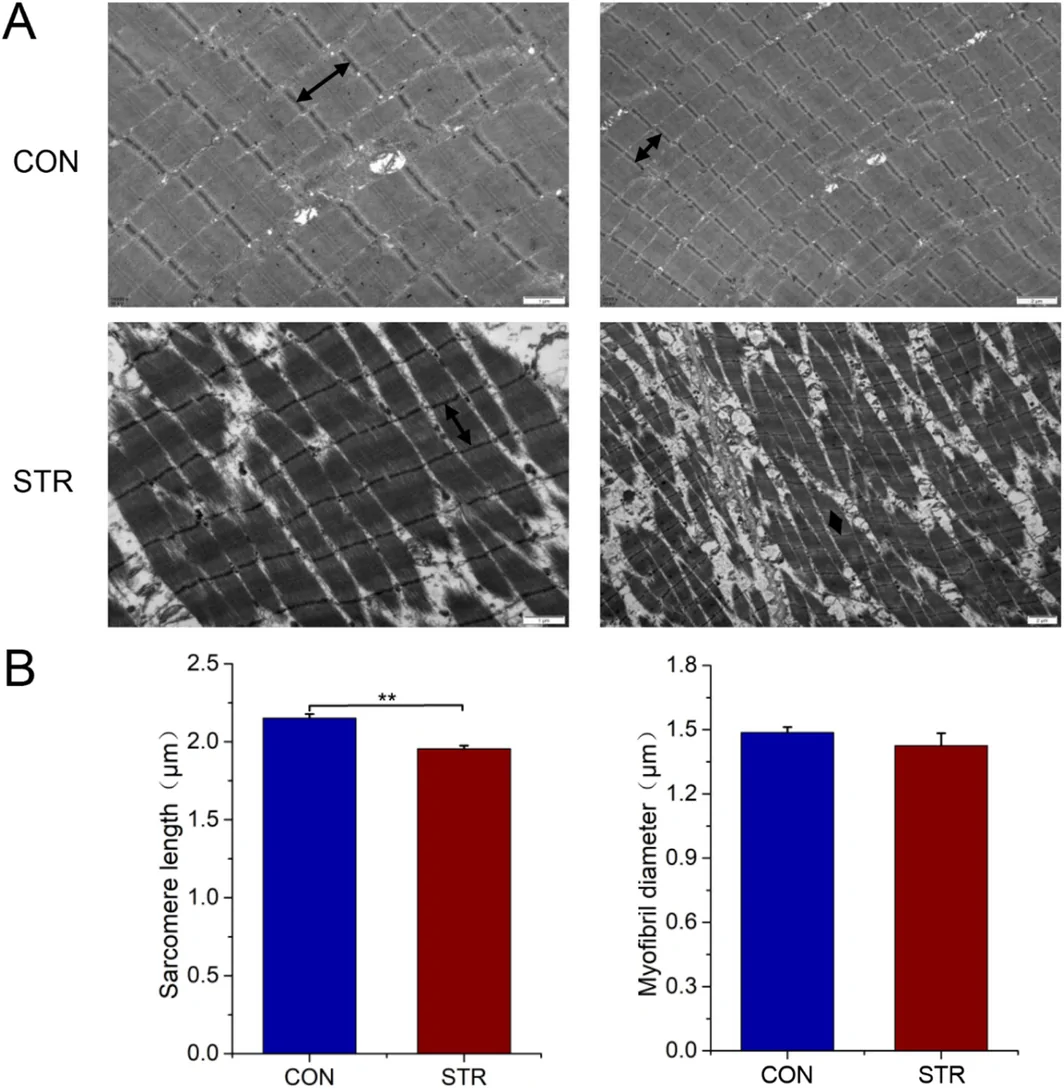
Transport stress alters sarcomere structure in beef. (A) Transport 0 h (CON) and transport 15 h (STR) myofibrils and sarcomeres under transmission electron microscopy. The scale bars represent 1 μm (left panel) and 2 μm (right panel). (B) Statistical analysis of sarcomere length and myofibril diameter. *p < 0.05; **p < 0.01; “↔” Represents a complete sarcomere.

### 3.2 Volatile Flavor Compounds Analysis

Raw meat has almost no aroma or taste, with a blood‐like taste. However, cooked meat forms its flavor characteristics through the complex interaction of flavor precursors in meat protein and fat components, resulting in volatile flavor compounds (Van Ba, Amna, and Hwang 2013). More than 700 volatile flavor compounds have been identified in beef, including aldehydes, alcohols, ketones, esters, furans, and pyrazines. These compounds alone or in combination give beef its unique aroma, flavor, and palatability, thereby affecting consumer acceptance. The oxidative degradation of linoleic acid produces hexanal and valeraldehyde, which contribute to the subtle aroma (Van Ba, Ryu, et al. 2013). Using GC‐IMS, we detected 53 volatile compounds, of which 42 were successfully identified by comparing their spectra with the GC‐IMS database. A total of 10 ketones, 11 alcohols, 15 aldehydes, 4 esters, 1 furan and acids were identified (Table 3). The PCA diagram (Figure 2A) and three‐dimensional diagram (Figure 2B) indicate that long‐distance transportation stress can lead to substantial changes in volatile flavor compounds. Overall, long‐distance transportation stress affected ketones, alcohols, and aldehydes. The specific manifestations were as follows: the content of 2,3‐butanedione, acetic acid, acetoin dimer, acetoin monomer, cyclopentanone, 1‐propanol, 2,3‐butanediol, 3‐octanol monomer, ethanol, benzaldehyde dimer, benzaldehyde monomer, ethyl acetate dimer, hexanal monomer, and octanal dimer in the STR group was lower than in the CON group (*p* < 0.01 or *p* < 0.05). The content of 2‐hexenal, acetone, 1‐pentanol polymer, and nonanal was higher than in the CON group (*p* < 0.01 or *p* < 0.05). Aldehydes are mainly formed by the oxidation of unsaturated fatty acids. Heptanal has a nutty flavor and fruit green fragrance; octanal has a characteristic odor of fruit and fat, and when diluted, it imparts a strong, fragrant aroma. Hexanal has apple aroma, leaf aroma, and subtle fragrance (Wang et al. 2021). Benzaldehyde has an aromatic aroma of almonds (Wolken et al. 2004).

**TABLE 3:** Changes of flavor substances in Holstein steers after transportation stress.

NO	Compound	Retention index	Retention time, s	Drift time, ms	Intensity (V)		*p*
					CON	STR	
Ketones							
1	2,3‐Butanedione	585.90	131.85	1.18	572.14 ± 18.64	475.01 ± 19.65	0.012
2	2‐Butanone	590.30	133.80	1.25	281.49 ± 11.67	350.07 ± 45.49	0.175
3	2‐Heptanone dimer	892.30	372.16	1.63	33.11 ± 3.52	33.90 ± 3.06	0.873
4	2‐Heptanone monomer	892.80	373.04	1.26	153.82 ± 24.77	171.17 ± 10.16	0.563
5	2‐Hexanone	792.90	262.55	1.50	107.21 ± 8.14	134.63 ± 1.08	0.110
6	2‐Pentanone	684.50	174.92	1.38	343.17 ± 24.99	372.90 ± 21.24	0.392
7	Acetoin dimer	732.30	211.56	1.33	3860.84 ± 570.24	192.91 ± 19.67	0.000
8	Acetoin monomer	734.30	213.19	1.06	2228.59 ± 183.76	707.56 ± 20.82	0.000
9	Acetone	522.00	103.94	1.11	7021.32 ± 75.21	8405.55 ± 293.57	0.033
10	Cyclopentanone	797.70	267.90	1.11	91.00 ± 6.906	72.11 ± 3.77	0.043
Alcohols							
11	1‐Butanol	672.40	169.63	1.18	505.57 ± 80.80	628.56 ± 54.17	0.235
12	1‐Pentanol dimer	778.40	248.34	1.51	706.89 ± 86.90	759.90 ± 59.59	0.633
13	1‐Pentanol monomer	778.40	248.34	1.26	1311.32 ± 80.49	1413.96 ± 77.79	0.395
14	1‐Pentanol polymer	776.20	246.58	1.81	34.14 ± 2.74	45.83 ± 5.90	0.039
15	1‐Penten‐3‐ol	694.00	181.03	0.95	195.16 ± 22.18	191.08 ± 16.21	0.883
16	1‐Propanol	572.60	126.06	1.12	376.83 ± 71.43	166.88 ± 15.68	0.017
17	2,3‐Butanediol	792.90	262.55	1.36	817.35 ± 51.33	636.92 ± 31.54	0.017
18	2‐Methyl‐3‐furanthiol	869.30	346.70	1.14	1390.55 ± 287.93	989.78 ± 142.51	0.241
19	3‐Octanol dimer	991.70	548.70	1.80	72.45 ± 4.80	75.44 ± 6.01	0.702
20	3‐Octanol monomer	991.70	548.70	1.41	2135.34 ± 249.21	1336.18 ± 38.38	0.013
21	Ethanol	530.30	107.58	1.04	10612.14 ± 528.51	8936.19 ± 325.69	0.022
Aldehydes							
22	2‐Heptenal (E)	958.70	490.14	1.25	88.26 ± 11.10	97.05 ± 9.97	0.578
23	2‐Hexenal	849.30	324.74	1.18	82.29 ± 5.71	116.32 ± 9.62	0.012
24	Benzaldehyde dimer	989.80	545.23	1.47	764.52 ± 85.27	522.09 ± 49.56	0.037
25	Benzaldehyde monomer	1002.80	569.55	1.16	1123.12 ± 109.97	783.64 ± 56.71	0.025
26	Butanal	598.70	137.46	1.29	442.98 ± 29.44	396.20 ± 55.56	0.474
27	Heptanal dimer	901.70	388.85	1.70	36.35 ± 4.75	33.69 ± 2.37	0.650
28	Heptanal monomer	902.20	389.73	1.33	237.50 ± 46.39	272.05 ± 13.46	0.529
29	Hexanal dimer	795.10	265.03	1.56	2069.98 ± 552.49	2493.44 ± 343.04	0.533
30	Hexanal monomer	795.90	265.90	1.25	2355.52 ± 114.50	1875.04 ± 122.29	0.019
31	Nonanal	1108.70	775.74	1.47	301.45 ± 13.05	359.15 ± 24.39	0.042
32	Octanal dimer	1012.80	588.96	1.82	81.22 ± 3.56	61.87 ± 2.12	0.001
33	Octanal monomer	1013.30	589.89	1.41	240.80 ± 32.53	279.82 ± 21.64	0.365
34	Pentanal dimer	695.50	182.25	1.42	69.51 ± 19.68	71.26 ± 11.96	0.938
35	Pentanal monomer	695.50	182.25	1.19	586.79 ± 60.23	608.18 ± 64.64	0.814
36	Phenylacetaldehyde	1043.10	648.07	1.26	60.25 ± 2.55	56.63 ± 3.01	0.379
Esters							
37	Ethyl acetate dimer	603.40	139.50	1.33	469.45 ± 37.41	329.95 ± 40.13	0.044
38	Ethyl acetate monomer	607.10	141.13	1.10	785.40 ± 29.27	748.40 ± 49.76	0.539
39	Ethyl hexanoate	1008.50	580.65	1.34	114.05 ± 17.17	113.96 ± 4.08	0.996
40	Isoamyl butyrate	1048.10	657.81	1.41	78.57 ± 2.92	74.78 ± 2.05	0.335
Furan							
41	2‐Pentylfuran	996.70	557.56	1.25	96.05 ± 16.74	99.31 ± 5.96	0.870
Acids							
42	Acetic acid	622.80	147.96	1.16	175.27 ± 32.09	72.36 ± 4.02	0.010

**FIGURE 2:**
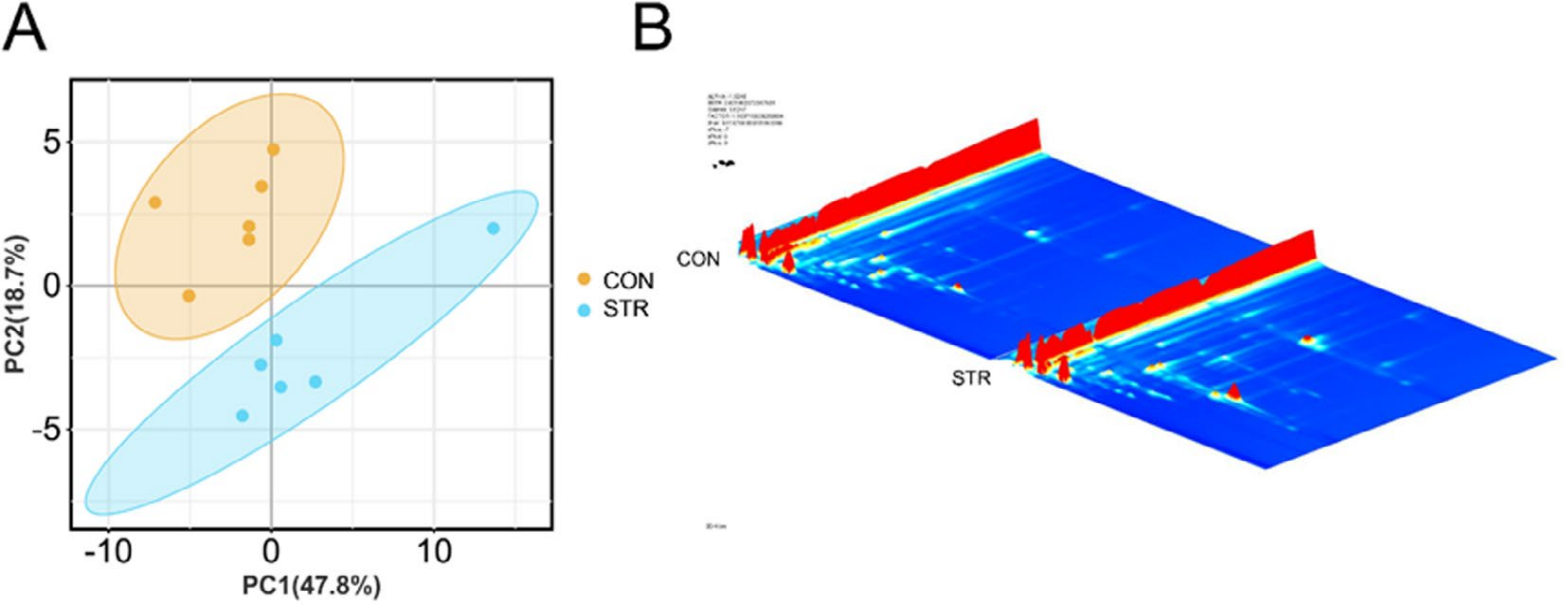
Transportation stress alters flavor compounds in beef. (A) Analysis of flavor substances using PCA. (B) Three‐dimensional topographic plots from CON and STR samples.

In our study, the reduction in the content of hexanal monomer, octanal dimer, benzaldehyde dimer, and benzaldehyde monomer may lead to a decrease in fruit green fragrance and other aromatic notes in meat. A compound may yield multiple signals or spots under conditions of high proton affinity or elevated concentration, which can be attributed to the sharing of protons or electrons among several molecules during the ionization process, leading to the formation of dimers or polymers (Wang et al. 2020). Since the monomer and the dimer belong to compounds of the same structure, only one calculation is required (Li et al. 2022). Alcohol, as an intermediate in the formation of meat flavor, is mainly produced through fat oxidation reactions, especially starting with n‐6 polyunsaturated fatty acids. Aldehydes can also be reduced to corresponding alcohol compounds (Lorenzo et al. 2013). Alcohols at high concentrations contribute to meat flavor, being herbaceous, woody, and fatty (Shi et al. 2018). Most ketones have high thresholds and have a small contribution to flavor characteristics, although some ketones are key intermediates in the production of heterocyclic compounds and play an important role in the formation of meat flavor (Edris and Fadel 2002). The 2,3‐butanedione and acetoin dimer imparts a cheese aroma to beef. Cyclopentanone has an important contribution to the aroma of beef during heating (Macleod and Ames 2010). Esters are the main components that form the aroma of fruits. In summary, our study indicates that the concentrations of several volatile compounds in beef—specifically hexanal monomer, octanal dimer, benzaldehyde dimer, benzaldehyde monomer, and ethyl acetate dimer—decreased significantly after long‐distance transport, which may potentially affect the flavor profile of the beef. On the other hand, transport stress arising from a combination of unfamiliar environments, unfamiliar cohorts, and vehicular vibration can induce physiological and biochemical changes in the animals' cells, thereby disrupting normal homeostasis (Gou et al. 2021). It should be noted that sensory evaluation was not conducted in the present study; therefore, future investigations may incorporate sensory assessment to further elucidate the effects of transport stress on beef flavor.

### 3.3 Overall Statistics for Transcriptomic and Proteomics Analysis

To further understand the underlying mechanisms through which transport stress affects meat quality and flavor, we performed transcriptomic and proteomic sequencing on the LL muscle. In transcriptional analysis, 49,320,549 and 48,101,475 original reads were obtained from the CON and STR groups, respectively; after quality control, 46,705,679 and 43,908,560 high‐quality clean sequences were obtained, respectively (Table S1). The sequencing quality was high enough to be used for subsequent quantitative analysis. Using transcriptome analysis, a total of 14,581 genes were identified in CON and STR, including 5976 unknown genes. Between CON and STR, 639 DEGs were obtained. Using CON as a control, 174 genes were upregulated and 465 genes were downregulated in the STR group (Figure 3A; Table S2). In addition, the results of hierarchical clustering showed that the expression profiles of genes from two different groups were distinguishable (Figure 3B).

**FIGURE 3:**
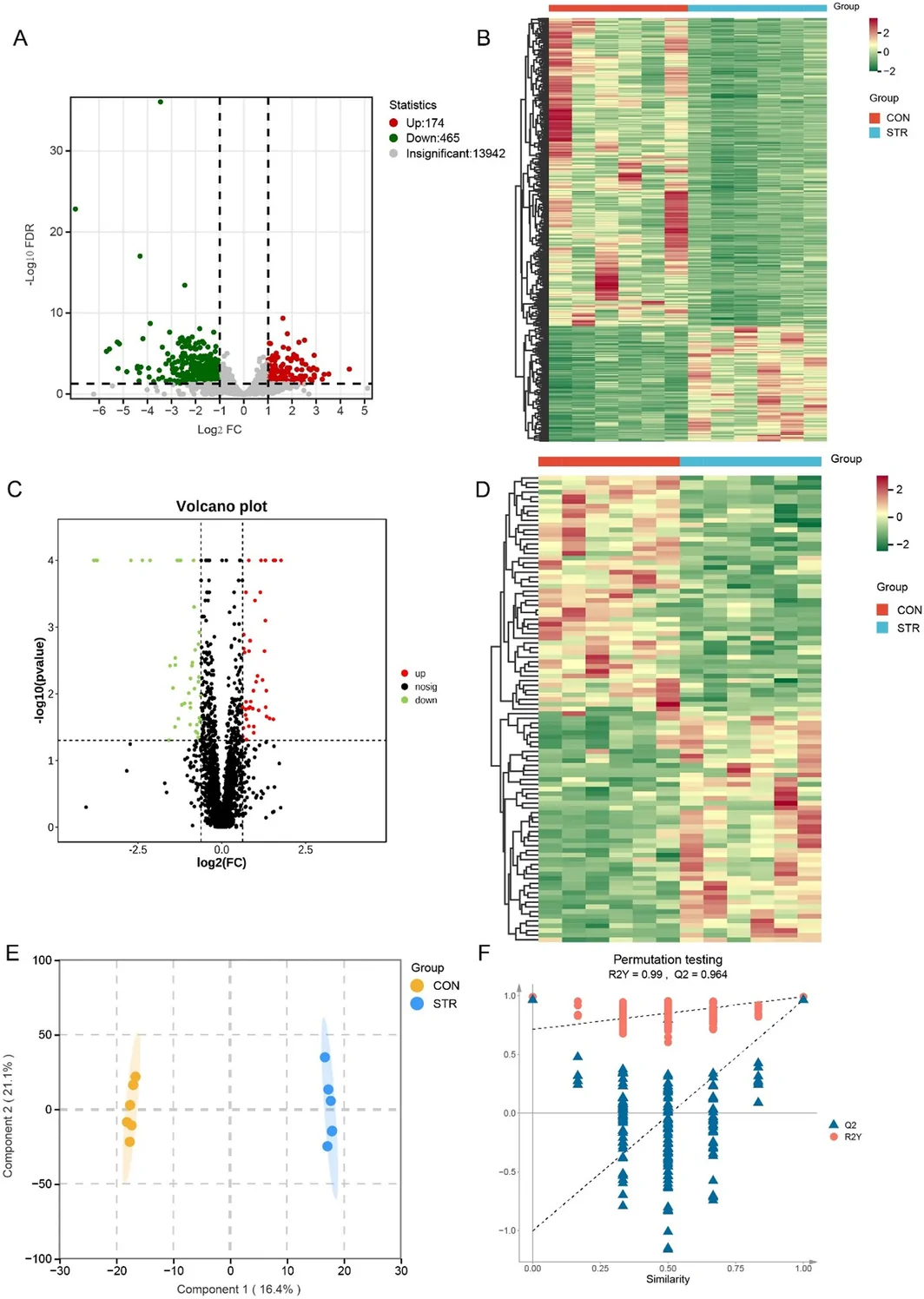
Differentially expressed genes (DEGs) and proteins (DEPs) in the CON vs. STR groups. (A) Volcano plot of DEGs; (B) Heat map of the DEG clustering analysis. (C) Volcano plot of DEPs. (D) Heat map of the DEP clustering analysis. OPLS‐DA analysis (E) and permutation test (F) of proteomic data.

A total of 2013 proteins were identified in both the CON and STR groups during proteomic analysis. Compared to CON, a total of 99 DEPs were detected in STR, including 48 upregulated proteins and 51 downregulated proteins (Figure 3C). Cluster analysis of DEPs indicated that the expression profiles of different histones were distinguishable (Figure 3D). Subsequent OPLS‐DA analysis of all quantified proteins revealed clear separation between samples from the two groups (Figure 3E). The permutation test (*n* = 200) was performed to evaluate the robustness of the OPLS‐DA model. The results showed that the R^2^Y and Q^2^ values of the original model were 0.99 and 0.964, respectively. All permutated Q^2^ values on the left were consistently lower than the original Q^2^ point, and the Q^2^ regression line intersected the y‐axis at a negative value, indicating that the model was not overfitted and possessed high predictive ability (Figure 3F).

### 3.4 Enrichment Analysis of Differentially Expressed Genes and Proteins in GO and KEGG

At the transcription level, the DEGs detected in the comparison between CON and STR significantly enriched 434 GO terms (*p* < 0.05; Table S3). In the category of biological processes, the DEGs were primarily enriched in “negative regulation of signal transduction”, “positive regulation of protein modification process” and “regulation of cell death”. In the category of cellular component, the DEGs were primarily enriched in “organelle inner membrane”, “extracellular matrix”, and “mitochondrial inner membrane”. In the category of molecular function, the DEGs were primarily enriched in “DNA‐binding transcription factor activity, RNA polymerase II specific”, “regulatory region nuclear acid binding” and “translation regulatory region DNA binding”. Figure 4A shows the top 10 GO terms with the highest number of DEGs for each category. In GO enrichment analysis, a greater number of significantly enriched DEGs/DEPs signifies higher biological relevance.

**FIGURE 4:**
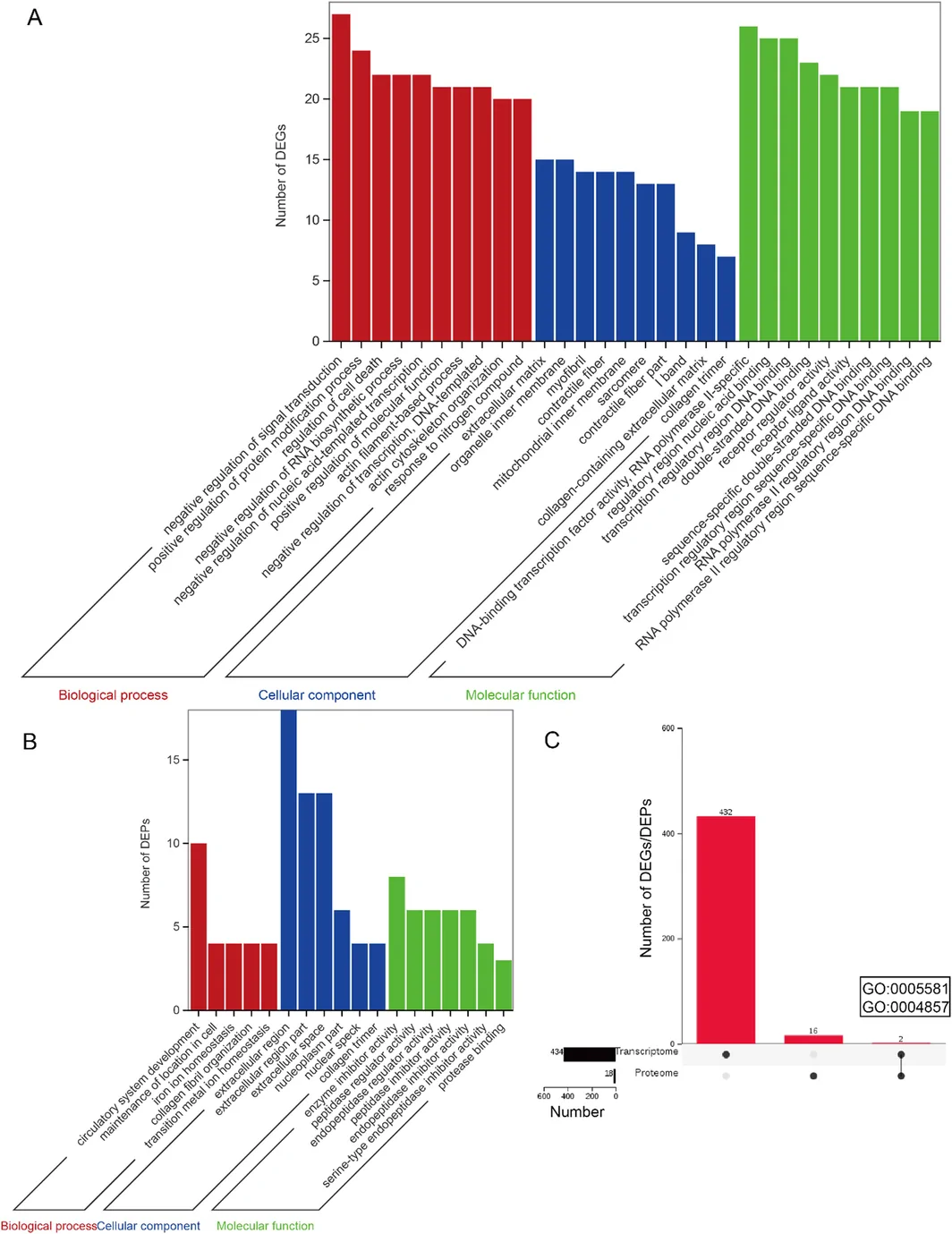
Functional enrichment analysis of differentially expressed genes (DEGs). (A) GO term enrichment for DEGs in the CON vs. STR groups; (B) GO term enrichment for DEPs in the CON vs. STR groups. (C) Venn diagram of GO terms enriched jointly by DEGs and DEPs.

At the proteomic level, CON enriched 18 GO terms compared to STR (*p* < 0.05; Table S4). In the category of biological processes, the five terms “circulatory system development”, “maintenance of location in cell”, “iron ion homeostasis”, “collagen fiber organization” and “transition metal ion homeostasis” were used more frequently. Under the category of cell components, the terms mainly included “extracellular region”, “extracellular region part”, “extracellular space” and “nucleoplasm part”. Under the molecular functional category, “enzyme inhibitor activity”, “peptidase regulator activity”, “endopeptidase regulator activity,” and “peptidase inhibitor activity” were mainly included (Figure 4B). It is worth noting that a cellular component (collagen trimmer; GO: 0005581) and a molecular function (enzyme inhibitor activity; GO: 0004857) were both enriched at the transcriptional and proteomic levels (Figure 4C).

KEGG pathway enrichment analysis was performed on DEGs and DEPs to identify biological functions that may be related to beef quality. At the transcriptional level, 58 enriched pathways were identified in the comparison of CON and STR (Table S5). The top 20 rich pathways are shown in Figure 5A. The most enriched pathways were “MAPK signaling pathway”, “Human papillomavirus infection” and “PI3K‐Akt signaling pathway”. Some of these pathways have been proven to be related to the biosynthesis of fatty acids, such as the “MAPK signaling pathway” and the “PI3K‐Akt signaling pathway”.

**FIGURE 5:**
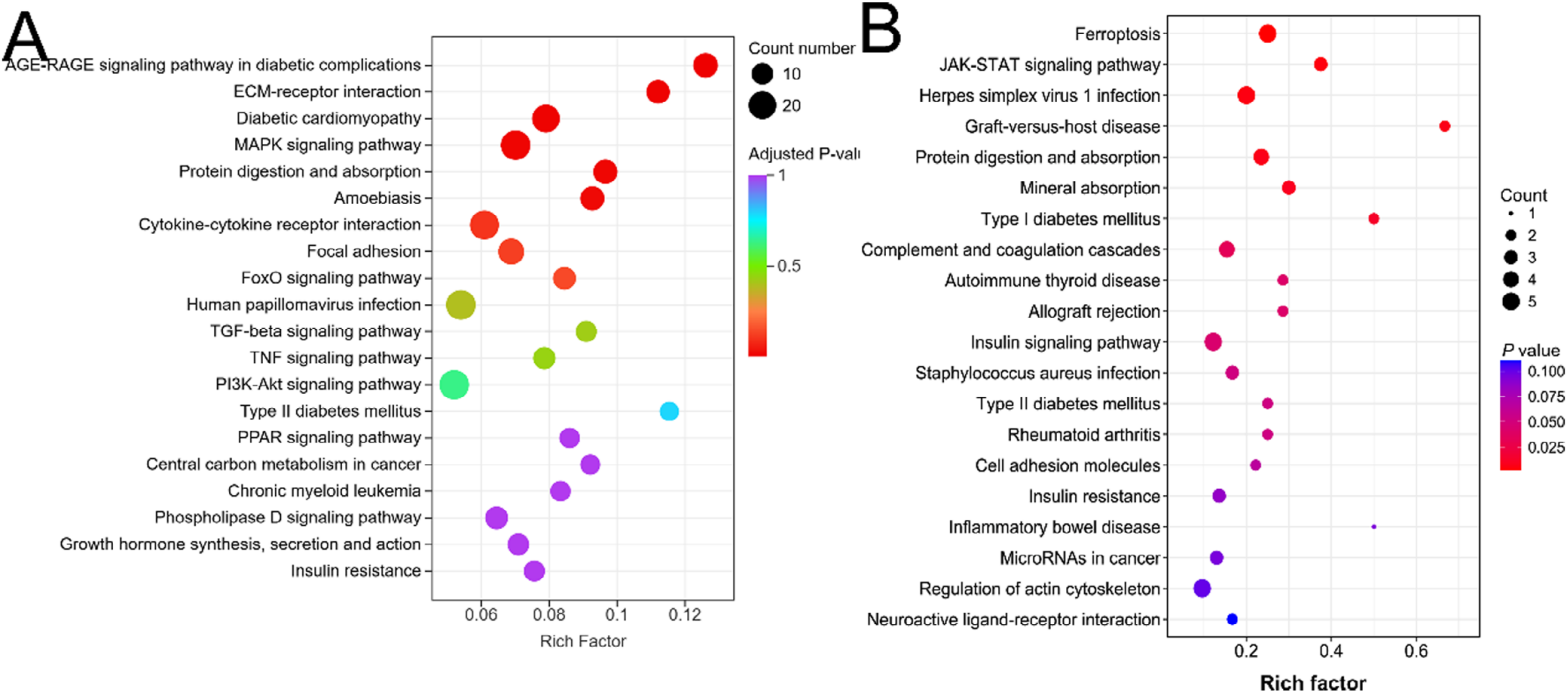
(A) Bubble chart of the top 20 pathways in KEGG analysis of DEGs. (B) Bubble chart of the top 20 pathways in KEGG analysis of DEPs.

At the proteomic level, DEPs compared between CON and STR were substantially enriched in 11 pathways (Table S6). The significantly enriched KEGG pathways were “Ferrotosis”, “Herpes simplex virus 1 infection”, “Insulin signaling path”, “Protein digestion and absorption”, “Complement and coagulation cascades”, “JAK‐STAT signaling path” and “Mineral absorption” (Figure 5B). Of the seven most enriched pathways, “Ferroptosis”, “JAK‐STAT signaling pathway” and “Mineral absorption” had the most enriched up‐regulated genes, while only 1 or 0 down‐regulated genes were enriched, indicating that the stress response caused by long‐distance transportation activated these three pathways and affected muscle properties. It has been previously reported that the occurrence of PSE meat may be associated with AMPK and HIF‐1 signaling cascades (Xing et al. 2018). In our current investigation, the major signaling pathways enriched in response to meat quality reduction caused by transport stress included PI3K‐Akt, MAPK, JAK–STAT, and ferroptosis. Liu et al. (2021) similarly demonstrated that the PI3K‐Akt signaling pathway and apoptosis participate in the physiological processes of postmortem muscle aging—for instance, by suppressing reactive oxygen species (ROS)‐induced apoptosis and inactivating apoptosis‐related proteins. Of note, apoptosis has been linked to the development of PSE pork. The novel metabolic pathways identified in our findings may provide a theoretical reference for further understanding the development of PSE or DFD meat.

Ferroptosis is a new type of programmed cell death that is iron‐dependent and distinct from apoptosis, necrosis, and autophagy. The main mechanism of ferroptosis is to catalyze the highly expressed unsaturated fatty acids on the cell membrane under the action of divalent iron or ester oxygenase, resulting in lipid peroxidation, thereby inducing cell death (Yang and Stockwell 2016). Generally speaking, the mechanism of lipid peroxidation is a free radical chain reaction, while reactive oxygen species (ROS) and iron (such as “free” iron in myoglobin) are the main initiators and main catalysts, respectively (Amaral et al. 2018). Studies have shown that lipid oxidation is not affected 24 h after death (Xu et al. 2011). However, transport stress causes iron ion imbalance and activates the ferroptosis pathway (Dixon et al. 2012). In order to balance iron ion homeostasis, some genes/proteins begin to accumulate in GO: 0055072 (iron ion homeostasis) and KO04630 (The Janus kinase/signal transmitters and activators of translation, JAK/STAT). The JAK–STAT signaling pathway represents a crucial signal transduction cascade downstream of cytokine receptors. Upon ligand binding to its cognate receptor, the receptor‐associated JAK kinases become activated. Subsequently, STAT proteins are activated through tyrosine phosphorylation by JAK kinases. This post‐translational modification facilitates their dimerization and subsequent translocation into the nucleus, where they modulate the expression of target genes (Imada and Leonard 2000). However, this does not seem to restore the balance of iron ions, leading to the occurrence of peroxidation, which produces some hexenaldehydes, which not only have toxic effects on the body (Sakai et al. 2000), but also affect the flavor of meat (Buckley et al. 1995). Lipid peroxidation can also lead to cytoplasmic loss, atrophy of connective tissue, transparent degeneration, and disorientation of muscle fibers (Monahan et al. 1993); and increase water loss and water loss rate (Ramirez and Cava 2007). Ma et al. (2023) and Xing et al. (2018) also reported that improper pre‐slaughter handling, such as transport stress, can lead to excessive generation of reactive oxygen species (ROS) and reactive nitrogen species (RNS) in post‐mortem muscle. These reactive radicals subsequently trigger protein oxidation, which deteriorates meat quality.

### 3.5 Combined Transcriptome and Proteome Analysis

Among the 639 DEGs, 563 did not display corresponding proteins in the proteomic data, which may be due to the relatively low sensitivity of proteomic testing. The proteins corresponding to 68 DEGs showed no marked difference in proteome (i.e., they were not DEPs). In the proteomic data, 77 DEPs whose coding genes had no expression changes between the CON and STR groups were identified (i.e., they were not DEGs), and 201 proteins had no corresponding transcripts. A total of eight DEGs/DEPs (BDH1, HMOX1, HSPB1, PFN2, COL1A1, FMOD, MYL3, and SLC16A3) showed changes at both the transcript and protein levels (Figure 6A).

**FIGURE 6:**
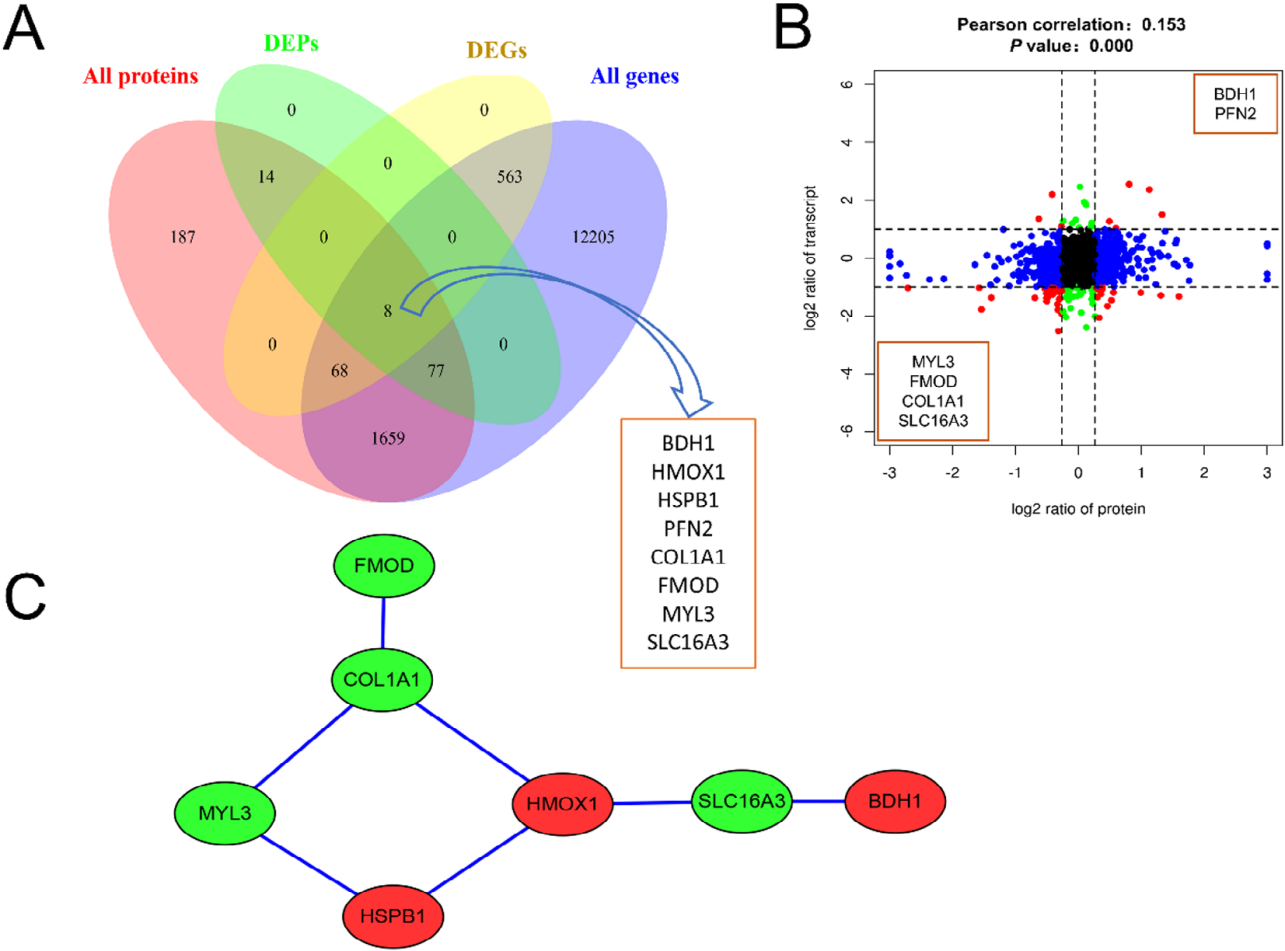
Joint analysis of the transcriptomes and proteomes. (A) Venn diagram of the numbers of all detected genes, DEGs, all detected proteins, and DEPs in the CON vs. STR groups. (B) Correlation between transcripts (*y*‐axis) and proteins (*x*‐axis) in the CON vs. STR groups. The *x*‐axis and *y*‐axis indicate the log_2_ fold‐change of proteins and log_2_ fold‐change of genes, respectively. Significant changes in expression are color‐coded: Blue, proteins only; green, transcripts only; red, both. (C) Protein–protein interaction (PPI) analysis showing the relationships of the target DEPs. Red indicates up‐regulated proteins, and green indicates down‐regulated proteins. More lines between two proteins represents more possible interactions.

We further conducted a comprehensive analysis of the transcriptome and proteome of CON and STR. The data were poorly correlated (Pearson correlation coefficient, *r* = 0.153). Transcriptome and proteomic data came from identical samples, such that analyzing DEGs and DEPs with the same expression pattern can help to mutually verify and explain gene expression regulation. The proteins in quadrants three and seven exhibited the same expression pattern as their transcripts (Figure 6B). Among the eight DEGs/DEPs, two DEGs/DEPs (BDH1 and PFN2 upward trend) and four DEGs/DEPs (COL1A1, FMOD, SLC16A3, and MYL3 downward trend) were included in quadrants three and seven, respectively (Figure 6B). To visualize the interactions among the co‐identified DEGs and differentially DEPs more clearly and intuitively, protein–protein interaction (PPI) network analysis was performed using the STRING database, and the resulting network was visualized with Cytoscape software. Red represents upregulated genes/proteins, and green represents downregulated genes/proteins (Figure 6C). The more lines there are between two genes/proteins, the more likely their interaction will be. The network analysis results showed that there was a close relationship between upregulated DEPs and downregulated DEPs. HMOX1 and COL1A1 (degree = 3) corresponded to key nodes in the network, followed by MYL3, HSPB1, and SLC163 (degree = 2). PPI indicated that upregulated DEPs directly or indirectly affected downregulated DEPs (Figure 6C).

The PPI diagram also showed that HMOX1 protein was an important node. The *HMOX1* gene may reduce fat production by regulating multiple signal pathways (Liu et al. 2018). The *HMOX1* gene is widely recognized as a highly responsive and reliable biomarker for physiological reactions to oxidative stress. Its primary role is to catalyze the degradation of heme, thereby generating biliverdin, which is subsequently converted to bilirubin—a molecule with potent antioxidant and free radical‐scavenging capabilities (Huang et al. 2025). The observed upregulation of HMOX1 in this experiment may represent a compensatory defense mechanism activated in response to transportation‐induced stress. Furthermore, heme degradation can negatively impact meat color. Marbling score is the distribution state of visible fat in muscle. The decrease in fat production leads to a decrease in marbling score. BDH1 (3‐hydroxybutyrate dehydrogenase 1) catalyzes the reversible reduction of acetoacetic acid to 3‐hydroxybutyric acid. BDH1 is the first ketolytic enzyme. The level of ketone bodies in BDH1‐deficient mice decreases substantially compared to normal mice during 24 h of fasting (Otsuka et al. 2020). Ketone bodies are the products of fat decomposition, and the production of ketone bodies from fat decomposition during starvation is a function of the body (Lee et al. 2021). In the present study, BDH1 was significantly upregulated, and we speculate that this may be associated with feed restriction in dairy bulls during transportation. Three of the four downregulated genes/proteins (COL1A1, FMOD, SLC16A3, and MYL3) found in the current study can have a positive impact on muscle development or meat quality. For example, as the growth rate increases, the expression of *COL1A1* also increases (Akit et al. 2018). Collagen is a major component of the connective tissue in muscle, and alterations in its content and structure influence meat tenderness and texture (Han et al. 2025). Type I collagen is the predominant form in muscular connective tissue, and the *COL1A1* gene plays a critical role in its synthesis (Song et al. 2021). TEM observation revealed that transport stress induced partial autolysis of myofibrils, which may be associated with downregulated *COL1A1* gene expression. FMOD, a member of the proteoglycan family, assists in extracellular matrix assembly and participates in myoblast differentiation by regulating the interaction between MSTN and type IIB activin receptors. In addition, FMOD also plays a crucial role in collagen fibrillogenesis, cell adhesion, regulation of cytokine activity, and inhibition of apoptosis (Lee et al. 2021). MYL3 (myosin light chain 3), a structural component of muscle, is involved in the development of myofibrils and myosin, and is expressed in slow‐twitch muscle fibers. MYL3 is believed to be related to the tenderness of different beef varieties and has a high expression level in tender meat (Malheiros et al. 2019). Studies have shown that the expression of MYL3 is downregulated in PSE pork, which may be associated with its specific fiber type characteristics (Zequan et al. 2022). In this study, transport stress induced myofibrillar contraction, resulting in shortened sarcomeres and increased muscle shear force, phenomena that were potentially linked to the downregulated expression of the *MYL3* and *FMOD* genes. The SLC16A3 gene, also known as monocarboxylate transporter 4, is responsible for the trans‐epithelial transport of lactate and short‐chain fatty acids (Dias et al. 2017). Research on its relationship with meat quality is relatively limited. Overall, transport stress induces alterations in the expression of genes/proteins associated with oxidative stress, ketone body metabolism, and the synthesis of collagen and myofibrils. This mechanism is arguably a primary reason for the observed changes in meat quality and flavor. Therefore, the involved genes/proteins represent potential biomarkers for assessing transport stress.

### 3.6 Validation of RNA‐Seq‐Based DEGs Using qRT‐PCR and Correlation Analysis

To verify the correctness of the transcriptional data, the gene expression levels of eight DEGS were detected using qRT‐PCR. The qRT‐PCR results indicated that the expression patterns of these genes was the same as those obtained in the RNA‐seq data, which validates the RNA‐seq data and indicates that the transcriptome results are reliable (Figure 7A).

**FIGURE 7:**
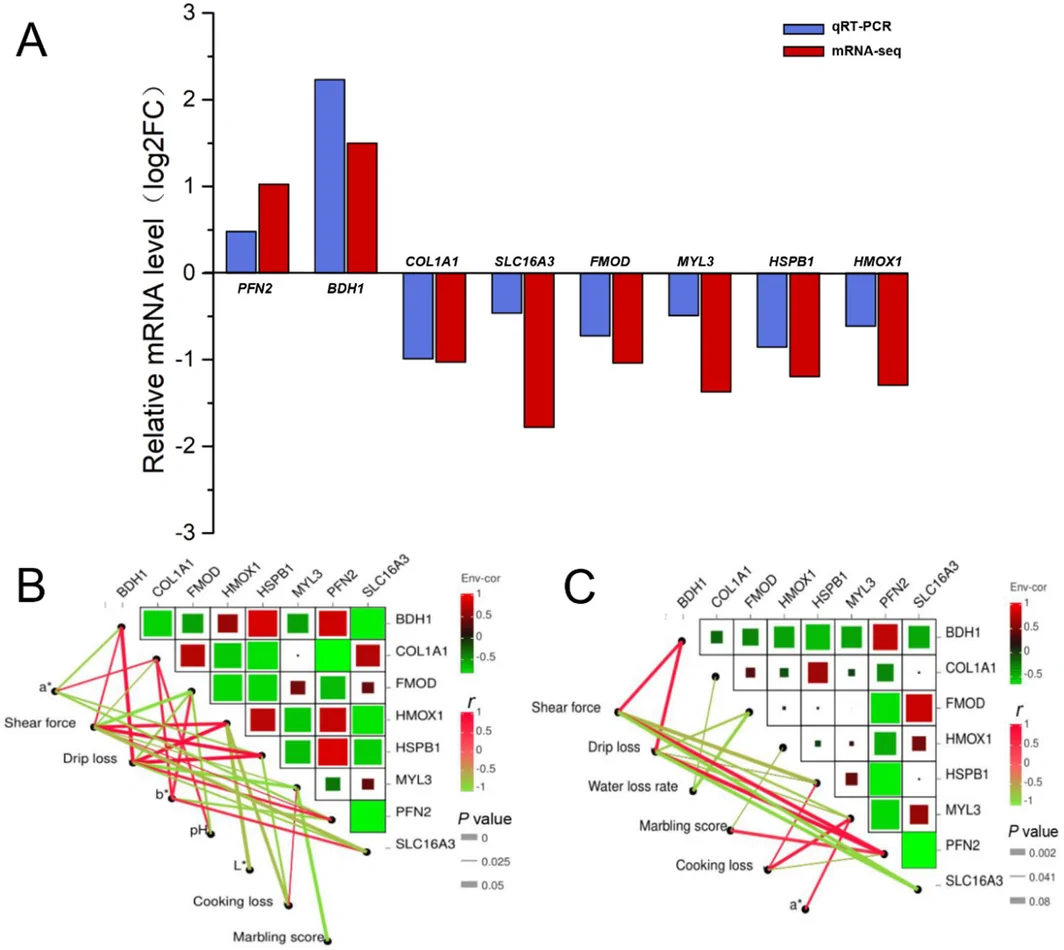
(A) Validation of RNA‐Seq by qRT‐PCR analysis. (B) Analysis of the association between phenotypes and DEGs. (C) Analysis of the association between phenotypes and DEPs.

To explore the relationship between these genes and phenotypes, the Pearson correlation coefficient between phenotypes and genes, and phenotypes and proteins was calculated, and |r| ≥ 0.6, and *p* ≤ 0.05 were of relevance. Visualization was conducted in the form of a network heat map. The two upregulated genes (*BDH1* and *PFN2*) had the strongest positive correlation with shear force and drip loss (Figure 7B). This correlation was more strongly expressed in proteins and phenotypes (Figure 7C). Of the six downregulated genes, *COL1A1*, *FMOD*, and *SLC16A3* showed the strongest negative correlation with shear force, while they showed the strongest positive correlation with b*. MYL3 had the strongest negative correlation with drip loss and marbling grade (Figure 7B). Overall, our study provides valuable insights into the effects of transport stress on meat quality and flavor in dairy bull calves, as well as the underlying molecular mechanisms. Furthermore, multiple studies have demonstrated that a period of lairage rest after long‐distance transportation can partially mitigate stress responses in livestock. In addition, in‐transit environmental factors including temperature, relative humidity, and stocking density exert notable stress impacts on animals. These unmeasured variables constitute a key limitation of the present experiment. Future systematic investigations are therefore warranted to clarify the combined effects of prolonged transport, varying post‐transport lairage durations, transit microclimates (temperature, humidity), and stocking density on animal physiological profiles (e.g., blood biochemical parameters) and final meat quality traits.

## 4 Conclusions

This article studied the effects of transportation stress on beef quality using transcriptomics and proteomics. Transport stress may impair beef quality, muscle fiber structure, and flavor by regulating the ferroptosis pathway and lipid peroxidation, and our data indicate that these effects are associated with concurrent changes in stress‐responsive genes/proteins (HMOX1, BDH1) and meat quality‐related genes/proteins (COL1A1, FMOD, MYL3). Our results provide important insights into the impact of transportation stress on beef quality, which may provide a new platform for studying the stress generated during transportation.

## Author Contributions

**Zhaomin Lei:** writing – original draft, funding acquisition, project administration, writing – review and editing. **Siyu Cheng:** conceptualization, investigation, writing – original draft, writing – review and editing, supervision. **Ruixin Yang:** resources, methodology, writing – review and editing. **Jinping Shi:** methodology, resources, writing – review and editing. **Zemin Li:** visualization, software, methodology, resources. **Wangjing Liu:** formal analysis, conceptualization.

## Funding

This research received funding from the Major Science and Technology Special Project of Gansu Province (25ZDNA008), the Modern Agricultural Industry Technology System Project of the Gansu Province Department of Agricultural and Rural Affairs (033‐034076), the Industry Support Project of Gansu Province (2024CYZC‐36), the Beef Cattle Quality Fattening Project of Gansu Province (GSA‐XMLZ‐2021‐01), and the Major Science and Technology Special Project of Gansu Province (GSSLCSX‐2020‐1).

## Ethics Statement

The animal study was reviewed and approved by the Institutional Animal Care and Use Committee of the Gansu Agricultural University under permit number No. GSAU‐Eth‐AST‐2022‐035.

## Conflicts of Interest

The authors declare no conflicts of interest.

## Supporting information


**Table S1:** Transcriptome sequencing data matching rate.
**Table S2:** Genes and corresponding read counts identified via transcriptomic profiling.
**Table S3:** GO terminology enriched with DEGs.
**Table S4:** GO terminology enriched with DEPs.
**Table S5:** Pathways where DEGs are significantly enriched.
**Table S6:** Pathways where DEGs are significantly enriched.

## Data Availability

All sequencing data are available through the NCBI Sequence Read Archive (Bio Project ID: PRJNA947491). The raw DIA proteomic data and processed quantitative results generated in this study are publicly available in the iProX database (https://www.iprox.cn/) with the accession ID PXD080786.
